# Advances in Understanding the Interplay between Dietary Practices, Body Composition, and Sports Performance in Athletes

**DOI:** 10.3390/nu16040571

**Published:** 2024-02-19

**Authors:** Alexandra Martín-Rodríguez, Pedro Belinchón-deMiguel, Alejandro Rubio-Zarapuz, Jose Francisco Tornero-Aguilera, Ismael Martínez-Guardado, Carlota Valeria Villanueva-Tobaldo, Vicente Javier Clemente-Suárez

**Affiliations:** 1 Faculty of Sports Sciences, Universidad Europea de Madrid, 28670 Villaviciosa de Odón, Spain; sandra.martin.rodriguez8@gmail.com (A.M.-R.); alejandro.rubio.z@hotmail.com (A.R.-Z.); vctxente@yahoo.es (V.J.C.-S.); 2 Faculty of Biomedical and Health Sciences, Department of Nursing and Nutrition, Universidad Europea de Madrid, 28670 Villaviciosa de Odón, Spain; pedro.belinchon@universidadeuropea.es; 3 Faculty of Health Sciences, Camilo José Cela University, C. Castillo de Alarcón, 49, Villafranca del Castillo, 28692 Madrid, Spain; imartinez@ucjc.edu; 4 Faculty of Biomedical and Health Sciences, Universidad Europea de Madrid, 28670 Madrid, Spain; c.vtobaldo@gmail.com; 5 Grupo de Investigación en Cultura, Educación y Sociedad, Universidad de la Costa, Barranquilla 080002, Colombia

**Keywords:** athletes, dietary practices, body composition, sports performance, nutrition interventions, training adaptations, optimal nutrition, athletic success

## Abstract

The dietary practices of athletes play a crucial role in shaping their body composition, influencing sports performance, training adaptations, and overall health. However, despite the widely acknowledged significance of dietary intake in athletic success, there exists a gap in our understanding of the intricate relationships between nutrition, body composition, and performance. Furthermore, emerging evidence suggests that many athletes fail to adopt optimal nutritional practices, which can impede their potential achievements. In response, this Special Issue seeks to gather research papers that delve into athletes’ dietary practices and their potential impacts on body composition and sports performance. Additionally, studies focusing on interventions aimed at optimizing dietary habits are encouraged. This paper outlines the key aspects and points that will be developed in the ensuing articles of this Special Issue.

## 1. Introduction

The realm of sports performance represents a dynamic interplay of various factors, among which dietary practices and body composition stand as pivotal elements [1]. In this line, the importance of nutrition in sports is well established, with a growing body of research underscoring its role in enhancing athletic performance, improving recovery, and reducing the risk of injury and illness [2]. However, the one-size-fits-all approach is rapidly becoming obsolete, as individualized nutrition strategies tailored to specific sports, physiological demands, and personal health and fitness goals gain prominence [3].

Body composition, an athlete’s proportion of fat, muscle, and bone, is another critical factor in sports performance [4]. The relationship between body composition and performance is complex and sport specific [5]. For instance, while leaner body composition may benefit endurance athletes, sports requiring explosive power or strength may necessitate a higher muscle mass [6]. This Special Issue aims to dissect these relationships, providing insights into how athletes can optimize their body composition for their specific sporting demands. Furthermore, this Special Issue addresses the nutritional requirements for different sports. The energy and nutrient needs of an athlete vary significantly across different sports disciplines. Endurance sports, such as long-distance running, have different nutritional demands compared to strength-based sports like weightlifting [2]. Understanding these differences is crucial for developing effective dietary strategies that support the unique needs of each athlete.

Evaluating the current dietary practices of athletes is a multifaceted endeavor that extends beyond mere observation of food intake. It involves a deep dive into the intricacies of athletes’ nutritional habits, encompassing the identification of common deficiencies or excesses that could impact performance [7]. This comprehensive evaluation is crucial, as it lays the groundwork for developing more effective and personalized nutritional strategies. By understanding the real-world dietary patterns of athletes, including their specific nutritional gaps and excesses, sports nutritionists and dietitians can tailor interventions that address these unique needs [8]. This approach is not only about enhancing performance but also about ensuring the overall health and well-being of the athlete. The complexity of this task is compounded by the diverse range of sports, each with its specific nutritional demands, making the role of individualized dietary assessment and planning even more critical [9]. The factors influencing nutritional choices among athletes are as diverse as the sports themselves. Personal preferences, cultural backgrounds, and the accessibility of certain foods play significant roles in shaping an athlete’s diet [10]. Moreover, an athlete’s knowledge about nutrition, or lack thereof, can greatly influence their food choices. This Special Issue delves into these varied influences, providing a comprehensive overview of how factors like cultural dietary norms, availability of food resources, personal taste preferences, and nutritional education impact an athlete’s dietary decisions. Additionally, the goals of the athlete, whether they are health-related or performance-oriented, also steer their nutritional choices. Understanding these multifarious factors is essential for developing effective nutritional strategies that are not only scientifically sound but also culturally sensitive and personally appealing to athletes.

Nutrition’s role in training adaptations is a topic of paramount importance. The appropriate balance of macronutrients (carbohydrates, proteins, and fats) and micronutrients (vitamins and minerals) is crucial for optimizing athletic performance [11]. This balance influences various aspects of an athlete’s training, from enhancing endurance and strength to promoting efficient muscle recovery and growth [11]. The latest research in sports nutrition provides insights into how different dietary components can be optimized to support specific training adaptations [12]. For instance, the role of carbohydrates in energy provision and the importance of protein in muscle repair and growth are well documented [13]. Additionally, this Special Issue explores how micronutrients and hydration strategies contribute to reducing fatigue and enhancing overall training efficiency. This comprehensive examination of nutrition’s role in training adaptations underscores its significance in an athlete’s overall training regime. Optimal nutrition for recovery is another critical aspect covered in this Special Issue. Recovery is not just a passive process of rest but an active phase where nutrition plays a key role. The right nutritional strategies during the recovery phase can significantly enhance the repair and rebuilding of muscle tissues and replenish glycogen stores [14]. This section of this Special Issue delves into the roles of various nutrients, such as proteins for muscle repair, carbohydrates for glycogen replenishment, and electrolytes for fluid balance, in the recovery process. Understanding these nutritional needs is crucial for developing effective recovery strategies that help athletes return to training and competition more quickly and effectively [14].

The impact of dietary practices on an athlete’s long-term health is a topic that extends beyond the realm of performance enhancement [15]. The long-term health implications of an athlete’s diet are profound, with potential effects on cardiovascular health, bone density, immune function, and overall well-being [16]. This Special Issue explores the complex relationship between nutrition, health, and sports performance, emphasizing the importance of a balanced diet not just for short-term gains in performance but also for long-term health and quality of life. Interventions to improve dietary practices among athletes are essential for optimizing both performance and health outcomes. This Special Issue discusses various strategies to enhance athletes’ dietary habits, including educational programs to increase nutritional knowledge, personalized nutrition planning to cater to individual needs, and the use of technology for monitoring and improving dietary habits. These interventions are designed to empower athletes with the knowledge and tools they need to make informed dietary choices that support their performance and health goals.

**Figure 1:**
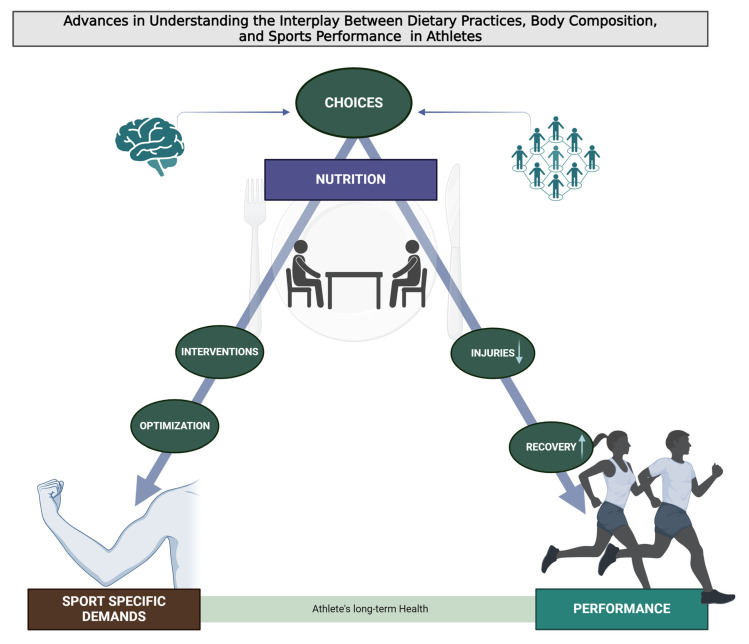
Graphical abstract of this comprehensive exploration of the complex interplay between dietary practices, body composition, and sports performance.

Then, this narrative review offers a comprehensive exploration of the complex interplay between dietary practices, body composition, and sports performance (Figure 1). It provides a blend of theoretical insights, practical strategies, and future perspectives, aiming to inform and inspire athletes, coaches, nutritionists, and researchers in the field of sports nutrition. This Special Issue underscores the multifaceted nature of sports nutrition, highlighting its critical role in enhancing athletic performance, supporting training adaptations, aiding recovery, and promoting long-term health.

Thus, we employed a thorough approach to gather pertinent literature, aligning with methodologies used by previous researchers in the field. Our comprehensive search strategy encompassed not only traditional academic databases but also extended to grey literature and consultations with subject matter experts. Key databases such as PubMed, Scopus, Embase, Science Direct, Sports Discuss, ResearchGate, and the Web of Science were utilized, alongside platforms like Google Scholar, to access a wider range of materials, including those not peer reviewed.

Our literature search was meticulously structured, employing keywords in line with MeSH standards such as “dietary practices”, “body composition”, “sports performance”, “athletic nutrition”, “performance-enhancing diets”, “sports-specific nutrition”, “nutritional deficiencies in athletes”, and “dietary interventions in sports”. This strategy was designed to comprehensively cover publications from 1 December 2013 to 15 December 2023 that are pertinent to the focus of our review. A team of seven seasoned authors was involved in screening the titles and abstracts of all gathered manuscripts. We established inclusion criteria centered on the relevance to the review’s theme, scientific integrity, and alignment with the subject of athletic performance and nutrition. Manuscripts that fell outside our specified timeline, were not in English, or were irrelevant to our focused area of research were excluded. This rigorous selection process was crucial in ensuring that only high-quality and relevant studies were included in our review. The same team was responsible for the critical task of data extraction and synthesis from the chosen studies. Each study was independently reviewed, and its findings were integrated into a coherent narrative. This approach allowed us to offer a comprehensive and systematic overview of the current understanding in the field, presenting a nuanced and in-depth perspective on the complex interplay between dietary practices, body composition, and sports performance, and exploring the implications for athlete health and performance optimization.

## 2. Body Composition and Performance Relationships

Understanding the relationship between body composition and performance across various sports disciplines is a complex yet crucial aspect of sports science. In aerobic sports such as long-distance running and cycling, a leaner body composition is often advantageous for efficiency and endurance. Knechtle, Wirth, and Knechtle (2010) found a significant correlation between lower body fat percentage and higher performance in endurance runners, emphasizing the importance of lean mass in endurance-based activities [17]. Similarly, Legaz and Eston (2005) demonstrated that triathletes with lower body fat percentages exhibited better race times, particularly in running segments [18]. In the context of long-distance running, the role of body composition extends beyond mere fat percentage. A study by Beattie, Kenny, Lyons, and Carson (2014) explored the impact of skeletal muscle mass on endurance performance [19]. They found that higher skeletal muscle mass, particularly in the lower body, was associated with improved running efficiency and performance. [19] This suggests that while lower body fat is advantageous, maintaining adequate muscle mass is also crucial for endurance athletes. Moreover, the distribution of body fat plays a role in endurance sports. A study by Knechtle, Wirth, and Knechtle (2010) also noted that not just the quantity, but the distribution of adipose tissue, impacts endurance performance [17]. Athletes with lower central body fat—or fat stored around the abdomen—tended to perform better in endurance events, indicating the importance of body fat distribution in addition to overall body fat percentage [17].

In cycling, where both endurance and power are essential, body composition significantly influences performance. A study by Menaspà, Quod, Martin, Peiffer, and Abbiss (2015) highlighted that a lower body fat percentage and higher lean body mass were indicative of higher power output in cyclists [20]. This underscores the need for a balanced approach to body composition, where lean mass must be optimized alongside minimizing excess body fat. Furthermore, the role of body composition in swimming, another aerobic sport, has been explored by Sharp et al. (2017), who found that swimmers with lower body fat percentages and higher lean body mass ratios exhibited better performance [21]. This aligns with the general trend in aerobic sports where efficiency, buoyancy, and power are enhanced by optimal body composition. These studies collectively emphasize that in aerobic sports, the goal of achieving a leaner body composition must be balanced with maintaining sufficient muscle mass. This balance is crucial not only for performance enhancement but also for injury prevention and long-term athlete health. The challenge for athletes and their support teams lies in crafting training and nutritional strategies that support the development of an optimal body composition tailored to the specific demands of their sport.

In anaerobic sports, where quick, explosive movements are essential, the importance of muscle mass and power cannot be overstated. This is particularly evident in sports like sprinting, where every millisecond counts, and high-intensity interval training, where short bursts of maximum effort are required. The relationship between muscle mass and sprint performance has been extensively studied. On this line, a research study by Seitz, Reyes, Tran, Saez de Villarreal, and Haff (2014) found that increases in lower body strength were significantly correlated with improvements in sprint performance [22]. This study underscores the importance of developing lower body strength for athletes involved in sprinting and similar high-intensity, short-duration sports.

Moreover, the type of muscle fibers plays a crucial role in anaerobic performance. Fast-twitch muscle fibers, known for their quick response and high-power output, are more prevalent in successful sprinters. A study by Egan and Zierath (2013) highlighted the significance of these muscle fibers in anaerobic activities [23]. They noted that athletes with a higher proportion of fast-twitch fibers tend to perform better in sports requiring short, intense bursts of energy [23]. In addition to muscle mass and fiber type, muscle power is a critical component of anaerobic performance. A study by Mero, Komi, and Gregor (1992) demonstrated that muscle power, particularly during the initial phase of sprinting, significantly influences overall sprint performance [24]. This finding suggests that training aimed at increasing explosive power can be beneficial for athletes in anaerobic sports.

Furthermore, the role of body composition in high-intensity interval training (HIIT) has been explored in recent research. A study by Boutcher (2011) found that HIIT can lead to significant reductions in body fat, particularly visceral fat, while improving muscle power and endurance [25]. This indicates that HIIT not only benefits athletes in terms of performance but also positively impacts body composition. These studies collectively highlight the multifaceted nature of body composition in anaerobic sports. While a higher muscle mass is beneficial, the type of muscle fibers and the ability to generate power quickly are equally important. Training programs for athletes in these sports need to focus not just on increasing muscle size but also on enhancing muscle quality and power output. This approach ensures that athletes can perform at their peak during the high-intensity demands of their sports.

In sports demanding explosive strength, the composition and quality of muscle tissue are paramount. A study by Comfort et al. (2012) demonstrated that in power athletes, muscle hypertrophy, particularly in fast-twitch muscle fibers, is strongly correlated with enhanced performance [26]. These fibers are known for their rapid force generation, making them crucial in activities requiring sudden, intense bursts of power. Similarly, Stone et al. (2003) found that muscle strength, particularly in the lower body, is a key predictor of sprint performance, highlighting the importance of muscle mass and strength in sports requiring quick, powerful movements [27]. Furthermore, the role of body composition in explosive strength and power extends beyond muscle characteristics. Fat mass, or the lack thereof, also plays a significant role. A lower body fat percentage can contribute to a higher power-to-weight ratio, which is crucial in sports where body weight must be propelled or moved rapidly. This was illustrated in a study by Zaras et al. (2013), which found that lower body fat percentages were associated with improved performance in weightlifting and jumping tasks [28].

## 3. Nutritional Requirements for Different Sports

In contemporary sports, an athlete’s preparation for competition is incomplete without a diet tailored to their specific training needs. A diet providing adequate caloric intake, inclusive of proteins, carbohydrates, and both micro and macronutrients, must align with the unique demands of each sport. Factors such as the athlete’s position on the field, the duration and type of sport, and adaptations to training dictate the energy system utilized by the athlete. To avoid performance-related issues and recovery challenges due to malnutrition and/or dehydration, as well as electrolyte imbalances, it is essential to design a nutritional plan for each specific phase in an athlete’s career. This plan should consider the varying requirements during competitive periods, training, or off-season, each with its distinct characteristics [29]. Nutrition in sports is planned, personalized, and periodized, aiming during training to promote recovery and adaptation, achieve optimal body composition, and minimize injuries and illnesses, thereby enabling athletes to train at the highest level. Therefore, the balance between carbohydrates, proteins, fats, and micronutrients should facilitate metabolic adaptations relative to the athlete’s constitution and training type [30]. Generally, athletes have a higher protein intake, essential for repairing and promoting muscle tissue growth [31,32]. Moreover, optimizing training and achieving peak competition results often involves the realm of supplementation. While the market offers a vast array of products, only a few are backed by solid scientific evidence [33,34]. Beyond these widespread practices among amateur and professional athletes, evidence suggests that the role of a sports nutritionist is indispensable for both performance and recovery, necessitating control over what, when, and how much to eat, including which supplements and fluids to consume, not just from a performance perspective but also for the athlete’s health [35].

Of course, nutritional requirements vary greatly depending on the physical activity. In endurance sports, Vitale and Getzin (2019) emphasize the rise in popularity of this sports category, leading to continuous revisions in sports nutrition and the supplementation industry [36]. The perpetual quest for top performance leads elite athletes to embrace new diets and/or supplements not yet supported by robust scientific evidence. The consumption of carbohydrates is well established in endurance athletes’ diets, as is the importance of total protein intake, particularly post exercise. Fat intake is increasingly being emphasized, especially for ultra-endurance athletes. The mandatory intake of fluids has been replaced with drinking according to thirst and sweat rates. Supplement use in endurance sports is widespread. Caffeine is perhaps the most common ergogenic aid, albeit with limitations due to side effects. Nitrates seem to enhance performance in non-elite recreational athletes, potentially increasing time to exhaustion, improving cardiorespiratory activity at the anaerobic threshold, and possibly VO2 max. Antioxidants might aid in recovery, beneficial in multi-stage events. The evidence for probiotics is less substantial, but urinary tract infections and gastrointestinal issues can be reduced with Lactobacillus and Bifidobacteria [33].

In sprinting, nutrition may not play as crucial a role as in endurance sports. However, the focus is on increasing muscle mass to generate more power. Here, again, protein intake is essential, along with an adequate proportion of carbohydrates and fats to ensure sufficient energy support [37]. Sprinters engage in intense activities lasting between 10 and 60 s, usually with sufficient recovery time between competitions to avoid depleting muscular energy stores. Nutritionally, the emphasis is on gastrointestinal well-being and avoiding weight gain during competitions. Pre-race intake should include 1 to 2 g/kg of body mass of carbohydrates 1 to 4 h before the event. Recovery should focus on easily digestible proteins and carbohydrates, and antioxidant intake in solid and liquid forms. Ergogenic aids in sprinting, such as bicarbonate or caffeine, must be thoroughly tested in training for their buffering effects [38].

In strength and power sports, the focus is on the consumption of protein-rich foods and essential amino acids in training, predominantly involving anaerobic muscle work [39]. Nutritional requirements in strength sports remain controversial due to their various subtypes: strength–endurance, power (speed–strength), and maximal strength [40]. There is a consensus on higher protein intake than recommended for strength or power athletes, regardless of their training level [41]. Arciero et al. (2015) reiterated the necessity for performance training to go hand-in-hand with sports nutrition [42]. Training for competition is known to cause muscle damage, pain, and inflammation. Many athletes adopt flexibility exercises and stretching to alleviate discomfort from exercise. Combining these restorative exercises with the intake of specific nutrients with analgesic and/or anti-inflammatory properties, such as ginger, turmeric, omega-3, or cherries, could be beneficial for athletes [43,44]. Although nutritional requirements vary based on the sport or the season, generally, steps should be taken to prevent undesirable health consequences under the supervision of specialists who also help athletes achieve peak performance. This involves prescribing the intake of highly nutritious foods and appropriate ergogenic aids, and continually assessing their effects on performance and health [43,44].

## 4. Optimizing Athlete Performance through Nutrition Insights

In recent times, there has been an increasing focus on the significance of adequate nutrition in athletic achievement. Athletes need tailored dietary habits to bolster their training, recuperation, and general well-being [45]. The nutritional state of athletes is pivotal in maximizing their performance. According to multiple organizations, excellent nutrition is essential for improving athletic performance and promoting post-exercise recovery wellness [35]. Despite this, athletes in weight-category sports or aesthetic sports often prioritize their weight and body image instead of a good quality nutrition plan [46]. Thus, this can impact their dietary preferences and lead them to adopt a specific nutrition plan. Besides internal factors, athletes are also affected by external influences like media and social pressures with regard to their nutrition choices [47,48].

Proper nutrition enables athletes to acquire the required fuel and essential nutrients for their physical endeavors, support muscle development and repair, and uphold overall health and wellness [35]. It is widely recognized that a well-rounded and healthy diet is crucial for enhancing both health and performance in the realm of sports. Nutrition not only influences physical abilities but also has an impact on athletes’ capacity to meet the specific functional demands of their respective sports [45,46]. An important aspect of assessing athletes’ present dietary habits involves evaluating their body composition [49], which plays a critical role in sports performance by influencing strength, power, and agility [50]. Nevertheless, despite the known advantages of proper nutrition for athletes, research indicates that many do not possess an adequate understanding regarding their distinct nutritional requirements for optimal performance [51].

Athletes’ level of nutrition knowledge is an important factor to consider when assessing dietary practices. Having a good understanding of nutrition is seen as crucial in improving athletic performance, while insufficient knowledge can negatively affect both the nutritional status and performance of athletes [52]. It is vital for athletes to grasp the importance of what, when, and how they should consume food and drink before, during, and after physical activity to enhance their performance and aid in optimal recovery [46]. Thus, numerous research studies have been carried out in the field of nutrition to explore these issues and assess the existing dietary habits of athletes. In this line, a study conducted by Cole et al. [53] explored the dietary practices of college football players and found that a significant portion of athletes consumed insufficient energy intake for their physical activity level and category. Similarly, Masoga et al. [54] reported that the dietary practices of athletes involved in the sport of soccer did not adhere to soccer nutritional guidelines established by the International Society of Sports Nutrition. Furthermore, Jusoh et al. [55] did not observe significant differences in the knowledge and practice of nutrition between sexes in handball. However, the results showed that nutrition knowledge had a significant but weak positive correlation with eating habits among athletes.

The absence of adequate nutrition evaluation and assistance for athletes is a matter of concern given the significant impact that nutrition professionals can have in enhancing athletic performance and ensuring proper fulfillment of nutritional needs [56]. Adequate nutrition is crucial not only for optimizing performance but also for minimizing the likelihood of injuries and illnesses among athletes. Despite the advantages and significance of appropriate nutrition for athletes, studies suggest that numerous athletes do not possess an adequate understanding about their distinct nutritional needs for their specific sport [50]. Therefore, a lack of understanding can result in misguided dietary choices and nutritional deficiencies. In general, assessing athletes’ current dietary habits involves examining how nutritional knowledge is applied to develop a customized nutrition plan that aligns with their physical activity, recovery needs, and overall well-being [57].

In conclusion, evaluating the current dietary practices of athletes is essential for identifying areas of improvement and providing them with proper guidance [58]. Moreover, evaluating athletes’ understanding of what and when they should be eating can help practitioners and coaches better understand their nutritional needs and the level of support required to optimize their performance [35,45,59].

## 5. Understanding the Dynamics of Nutritional Choices: Societal Influences

Nutrition-related decisions are crucial aspects of our daily routines, impacting bodily functions, energy levels, and overall health [60]. Our dietary preferences are not random; they are significantly influenced by our cultural background and societal structures, shaping culinary choices [61]. Economic factors also play a role, affecting the diversity, quality, and nutritional content of various dietary options [62]. Additionally, the evolving landscape of advertising and social platforms goes beyond simple information sharing, actively molding perceptions of appealing food choices [63]. Hence, nutritional decisions are interconnected with society, culture, economics, and media influences. Understanding this intricate web is vital for a comprehensive nutrition approach, enabling individuals to make well-informed and health-conscious dietary choices [64].

Initially, it is important to acknowledge that eating has transformed into a social and cultural event, expressing an individual’s and collective’s heritage and traditions [65]. Cultures impart unique culinary traditions, offering a diverse range of ingredients, preparation methods, and flavors [66]. Economic considerations become a powerful force, shaping the nutritional landscape based on individuals’ socioeconomic status [67]. While those in a better economic position enjoy diverse choices and nutrient-dense options, individuals with lower socioeconomic status face constrained choices, often opting for more economically feasible but less nutritious options, leading to health implications [68]. In the digital era, the interaction between media and marketing significantly influences dietary choices, shaping consumer preferences through social media narratives that extend beyond information dissemination [69]. This often directs consumers toward less nutritious options, perpetuating a cycle where convenience overrides health [70]. Furthermore, psychological factors, including emotional well-being, stress levels, and mental health, exert a significant impact on nutritional choices [71]. Positive mental states often prompt healthier choices, while stress may trigger cravings for less nutritious foods, revealing the complex interplay between emotions and dietary habits [72]. Personal preferences also play a pivotal role in shaping nutritional choices, as the spectrum of flavors and textures establishes the foundation for dietary habits, creating a distinctive gastronomic identity [73]. Additionally, convenience and accessibility are influential factors shaping the culinary choices of individuals in contemporary lifestyles [69]. In this context, easily obtainable food choices become a decisive force as individuals seek quick solutions to satiate hunger amidst hectic schedules [61,74]. Balancing convenience and nutritional considerations emerge as a critical challenge, requiring both individual awareness and societal initiatives that promote healthier convenience options [75]. Moreover, education and knowledge emerge as a guiding mechanism for individuals to make informed decisions. Access to accurate information enhances individuals’ ability to navigate the complexities of dietary information [76]. Informed individuals comprehend the broader context of food, contributing not only to individual well-being but also to the broader societal narrative around food, fostering a nutritionally literate society that embraces sustainable and health-conscious dietary habits [77,78].

Furthermore, a portion of the population faces specific health conditions and individualized constraints shaping nutritional choices. Allergies, intolerances, and unique dietary restrictions become guiding forces, compelling individuals to navigate their culinary choices with precision. These restrictions not only serve as pragmatic responses to health concerns but also shape one’s identity and daily decision-making process. Driven by health considerations, individuals find themselves compelled to choose foods that align with their physiological needs, transcending taste preferences or convenience [61]. This personalized approach to nutrition demands a heightened awareness of nutritional content, ingredient scrutiny, and alternative choices, requiring both individual agency and support from healthcare professionals [79]. Despite presenting challenges, health conditions and dietary restrictions open avenues for culinary creativity, transforming limitations into opportunities for discovering new flavors and textures [80]. The fusion of health-driven choices with culinary innovation not only meets nutritional needs but also enriches the culinary experience for individuals navigating the intricate terrain of dietary restrictions [80].

Moreover, when considering athletes, nutritional choices become pivotal, influencing factors from body composition to sports-specific capabilities, impacting performance and well-being [2]. The interplay between dietary practices, body composition, and sports performance is dynamic and multifaceted, shaped by various factors [58]. Athletes’ individualized needs, including age, gender, body composition, and metabolism, become crucial considerations [81]. Recent advances in nutrigenomics provide personalized dietary recommendations, unveiling how genetic makeup influences nutrient responses, allowing for tailored dietary recommendations [82]. Training intensity and type significantly impact energy expenditure and nutrient demands, with nutrient periodization allowing athletes to tailor nutritional intake to different training phases [83]. This ensures the optimization of energy levels, muscle recovery, and sustained peak performance throughout their competitive season [84]. Further, environmental factors, such as altitude and extreme temperatures, deeply influence athletes’ nutritional needs [85]. Strategic adjustments in hydration, electrolyte balance, and nutrient intake become pivotal tools in adapting nutrition [86]. Athletes, like those venturing into high altitudes, may require specific nutrient interventions for enhanced oxygen-carrying capacity, while those exposed to extreme heat must navigate hydration strategies to prevent dehydration and heat-related issues [87]. Additionally, athletes often harbor specific body composition goals intricately linked to the demands of their sport [58]. Advancements in body composition analysis technologies offer accurate tools for monitoring and adjusting dietary practices with surgical precision [88]. Precision nutrition becomes a guiding principle, ensuring dietary choices are calibrated to sculpt the optimal physique that complements the demands of their chosen discipline [89]. Furthermore, psychological, and emotional factors, including mental health and stress levels, play a role in nutritional choices for athletes [90]. Social and cultural influences shape dietary choices, and educational and access factors empower athletes to make informed dietary decisions, enhancing overall performance and well-being [2,10].

Then, factors affecting nutritional choices are diverse and interconnected, and recent advances in sports science and nutrition offer valuable insights for athletes to tailor their dietary practices. Analyzing athletes’ unique needs, training regimen, environmental conditions, psychological well-being, and cultural context holistically is essential. By addressing these factors comprehensively, athletes can make informed nutritional choices that contribute to their success and longevity in the competitive world of sports.

## 6. Optimizing Training with Nutritional Periodization in Sports: Adaptations

The adaptability to training is established through the interplay of training intensity, volume, and frequency [91,92]. The interconnection between nutrition and exercise plays a pivotal role in training adaptations. Indeed, dietary variations significantly influence training adaptation. Numerous possibilities exist within periodized nutrition or nutritional training, yet not all have equal scientific backing. Among the more accepted practices are training with varying carbohydrate availability, supplement use, and enhancement of intestinal function.

Athletes and trainers must remember that there are no miraculous methods, and that nutritional periodization should aim for specific objectives. Combining different methods can optimize nutritional training [79,84,93]. The goal of nutritional periodization is to enhance the body’s adaptation to training. Factors such as age, sex, physical condition, prior nutritional state, diet, and specific training factors like intensity, volume, timing, and rest periods are pivotal in determining the efficacy of nutritional training in producing training adaptations [94]. The vast diversity of athletic events, each with its unique bioenergetic, biomechanical, and structural demands, requires a wide range of periodized nutrition combinations. Thus, nutritional training strategies for proteins, supplements, or carbohydrates should be implemented and their complications observed [95]. For example, in protein intake, considering the total amount, type, timing, and pattern of intake is crucial. Proper application of these factors significantly impacts metabolic outcomes, thereby benefiting the athlete with more favorable training adaptations [86,90].

This is evident in the plasticity of skeletal muscle, which can adapt its metabolism based on the load and nutrient availability. Variability in macronutrient intake translates into changes in substrate and hormone density in the blood available to muscle and insulin-sensitive tissues. Consequently, the relationship between available substrates and exercise will determine the capacity to inhibit or activate different biochemical pathways, potentially impacting training adaptation [96].

In developing endurance, skeletal muscle exhibits training adaptations that are enhanced by nutritional periodization. It is posited that consuming fewer carbohydrates during appropriately planned periods and increasing protein intake can foster greater training adaptations [97]. Moreover, the adaptive response to endurance training can be heightened by factors such as glycogen depletion, caloric restriction, oxidative stress, among others. This increased stress at the molecular level elevates the quantity and activity of peroxisome proliferator-activated receptor gamma coactivator 1-alpha (PGC-1α), which can subsequently induce primary adaptive responses to resistance exercise: mitochondrial biogenesis, angiogenesis, and enhanced fat oxidation. Therefore, PGC-1α activation can be regulated considering nutritional training [98].

Nikolaidis et al. (2018) indicate that ultra-endurance athletes generally share similar habits with endurance athletes, except for a higher prevalence of vegan or vegetarian diets. They exhibit similar training duration parameters to those of endurance athletes [99]. However, carbohydrate intake is higher in ultra-endurance athletes, with the quality of these carbohydrates being crucial as it relates to higher exogenous carbohydrate oxidation and mitigating common gastrointestinal discomforts in ultra-endurance events [99]. Furthermore, pre-competition nutritional training in ultra-endurance athletes should emphasize fat utilization as fuel during exercise [99]. Thus, the nutritional adaptations to endurance training are broad and varied. Each adaptation has its specifics within nutritional periodization. Training at a moderate altitude (~1600–2400 m) presents a paradigm within endurance training. However, there are risks associated with insufficient energy availability and the need to facilitate higher iron intake in altitude training to avoid impeding adaptations. Additionally, oxidative stress caused by altitude training should be mitigated through antioxidant intake from foods, avoiding supplementation [86,100]. Therefore, combining metabolism, cardiovascular system, and muscular adaptations with appropriate nutrition and supplementation will result in enhanced performance for the endurance athlete [101].

Regarding high-intensity exercises, prevalent in many sports, supplementation becomes particularly interesting. The energy demand of anaerobic processes must be met to satisfy adenosine triphosphate (ATP) requirements. The amount of ATP that can be generated is determined by muscular phosphocreatine (PCr) content, buffering capacity, and the volume of contracting muscle mass. The latter two can be increased through anaerobic training. However, PCr is unaffected by training. There is solid scientific evidence on the benefits of dietary ergogenic aids like creatine and sodium bicarbonate or beta-alanine for increasing anaerobic capacity [102]. Yet, results on the effects of nutritional supplements are limited and sometimes perplexing. For instance, a study on five-day supplementation with one of the most used aids, creatine monohydrate, did not increase anaerobic power in trained wrestlers [103]. Sodium bicarbonate (0.3 g/kg of body mass) seems to be the most effective ergogenic aid, but undesirable side effects like intestinal discomfort are associated with such doses. Beta-alanine has been reported to cause paresthesia following a single intake. Therefore, it may be more advisable to seek the combined ergogenic effects of sodium bicarbonate and beta-alanine [104].

Despite the solid scientific backing for the co-ingestion of bicarbonate and beta-alanine, as well as creatinine with beta-alanine, in improving performance in high-intensity exercises, benefits might depend on the specific exercise and the athlete’s training state. Hence, unraveling the workings of these synergies requires further research [105].

## 7. Optimal Nutrition for Recovery

Engaging in any form of physical activity results in an elevation in metabolism and enhances the functioning of our body’s systems [106,107]. Athletes frequently employ several recovery tactics (biological, pharmacological, mechanical, and dietary) to enhance physiological responses and competitive performance to achieve optimal muscle recovery. Bonilla et al. showed the identification of a 4R’s strategy to maximizing post-exercise recovery based on a framework that relies on evidence. First, rehydration is a crucial process that varies depending on the athlete, environment, and sporting event. Second, refueling involves consuming carbohydrates, which not only replace glycogen reserves but also provide energy for the immune system and tissue repair. Third, repair by consuming high-quality protein and creatine monohydrate after exercising helps with the growth and repair of tissues and rest—nutrition before sleep has a rejuvenating impact that aids in the recovery of the musculoskeletal, endocrine, immunological, and nervous systems [31,41,108].

**Figure 2:**
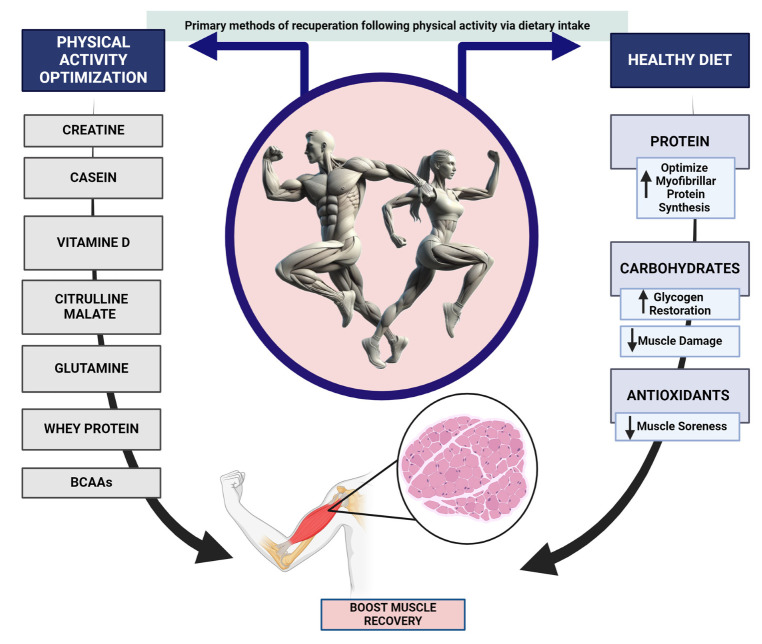
Primary methods of recuperation following physical activity via dietary intake.

Focusing on nutrition, extensive research has been conducted on proteins, amino acids, essential nutrients, and metabolic regulators (such as vitamins and minerals), which have consistently shown their significance and efficacy in promoting muscle repair [106,107]. This additional preparation could significantly and lawfully assist athletes in effectively supplementing their training to enhance their performance or attempt to expedite their journey to the top of their sport. Thus, the food plan plays a crucial role in stimulating muscle repair among the strategies applied. Thus, it is essential to optimize the intake of appropriate quantities of energy, nutrients, and fluids while establishing the proper frequency and timing about training and competition [84]. An expedited and more effective recuperation will enable athletes to engage in more intense exercise and exhibit a more favorable response to training, necessitating a sufficient nutritional intake [84]. Following an examination of the ergogenic supplements and nutrients most frequently utilized by athletes, this review will then concentrate our investigation on the supplementation utilized during various types of physical training (Figure 2).

### 7.1. Ergogenic Supplement and Muscle Recovery

Athletes are advised to use certain nutritional supplements that have been proven to enhance their workout performance. While some nutritional supplements may not directly enhance performance, they can potentially enhance an athlete’s health, exercise adaptation, and injury healing, hence aiding in more effective training and competition. The following is a brief overview about dietary supplements that have the potential to enhance overall health, facilitate exercise adaption, or expedite the recovery process.

Glutamine, along with other amino acids, promotes muscle growth by enhancing protein synthesis [103]. According to Street et al. [104], the addition of glutamine to a diet can reduce the intensity of the body’s inflammatory reaction following eccentric activity. Legault et al. [105] discovered that the addition of glutamine lowers muscular discomfort, suggesting a potential association with decreased muscle damage. More specifically, Cordova-Martinez et al. also reported the impact of glutamine supplementation on the recuperation process following eccentric muscle damage in elite basketball players. The findings indicate that glutamine supplementation leads to a reduction in the levels of muscle damage markers in the bloodstream [106,107,108,109,110]. This is accompanied by a proper equilibrium between the response of the hormones responsible for breaking down muscle tissue and those responsible for building it up, as well as the stability of the number of white blood cells [104,111,112].

Regarding vitamin D, Mieszkowski et al. [113] examined the anti-inflammatory properties of vitamin D in the context of exercise training. In a previous study, Choi et al. reported that vitamin D administration in an animal model greatly decreases inflammation caused by high-intensity exercise [114]. However, a recent meta-analysis examined the impact of Vitamin D on muscle recovery after exercise [115]. It was found that while Vitamin D appears to be beneficial in reducing muscle inflammation, its role in promoting post-exercise recovery by regulating the release of muscle biomarkers has yet to be proven [116]. Caballero- García et al. recommend that future trials incorporate cytokine measurements and consider variables such as extended administration duration or increased supplementing dosage. Vitamin D supplementation is used to restore and optimize blood levels of vitamin D from a practical standpoint. However, other studies employing similar techniques are required to obtain more robust information regarding post-exercise muscle recovery [116].

Creatine is extensively researched and widely used as an ergogenic supplement by athletes and weightlifters who aim to enhance their performance, boost exercise training effects, and reduce recuperation duration [117,118]. Research repeatedly demonstrates that using creatine supplements has a beneficial impact on both individual and repeated instances of brief, intense physical activities while also enhancing the body’s response to exercise training. In addition, the use of creatine supplements can accelerate the recovery time between periods of intense physical activity by reducing muscle damage and facilitating the faster restoration of lost muscle strength [119]. In fact, creatine monohydrate has the potential to accelerate recovery and adaptation to hard exercise, as well as recovery after periods of injury accompanied by prolonged inactivity. It may also improve cognitive processing and aid in the reduction in severity or promotion of recovery from mild traumatic brain injury (mTBI) [119].

Citrulline malate (CM) has been found to enhance aerobic energy production and increase phosphocreatine (PCr) levels during exercise recovery [120,121]. It also improves ammonia (NH3) elimination after exhaustive exercise [122,123], reduces muscle soreness following high-intensity resistance exercise (RE), and enhances performance in repeated bouts of high-intensity RE [124]. Ultimately, da Silva and colleagues demonstrated that the administration of CM at a dosage of 6 g taken 60 min before a workout does not enhance the process of muscle recovery following a single session of high-intensity resistance exercise in young adult men who are not trained in physical fitness. Consequently, it may be too early to suggest the use of complimentary CM as a performance-enhancing supplement to enhance muscle recovery following resistance exercise [125]. In fact, Gough et al. warned that the absence of beneficial outcomes from CM supplementation in the current body of research can be attributed to several factors. These include the testing protocols not primarily emphasizing aerobic energy contribution, the lack of consistency in exercise protocols for repeated testing, the strategy for administering CM (i.e., dosage and timing), and the recent identification of quality control problems with certain manufacturers’ citrulline/malate ratios [126].

In order to achieve maximum muscle protein synthesis and net protein balance, it is crucial to consume a source of protein after resistance exercise [127]. These factors are necessary to sustain muscular hypertrophy throughout training. Recent studies indicate that consuming a moderate amount (approximately 20–25 g) of quickly digested proteins that are high in leucine can enhance muscle protein synthesis [128]. However, in this regard, the recommendations for athletes to enhance muscle protein synthesis (MPS) through protein intake per serving are varied and depend on factors such as age and recent resistance training stimulus. It is generally recommended to consume 0.25 g of high-quality protein per kilogram of body weight or a total amount of 20–40 g [129]. This suggests that whey protein can be a beneficial supplement for individuals looking to optimize their recovery and adaptation to resistance exercise. Consuming 25 g or more as mentioned of whey protein following an evening session of resistance exercise showed a tendency to enhance overall net protein balance in the body throughout a 10 h overnight recovery period, compared to a control group that did not exercise [130]. Additionally, the whey protein supplement was moderately more effective than a post-exercise supplement containing the same number of calories from carbohydrates. Ingesting an extra 25 g of whey protein in the morning following exercise resulted in a higher overall protein balance in the body over the 24 h recovery period compared to a control group that did not exercise and received carbohydrate supplementation. The addition of whey protein was found to increase overall body anabolism, leading to improved recovery and exercise performance following a strenuous resistance workout [130]. Regarding this matter, it has been emphasized that the supplementation of branched-chain amino acids (BCAAs) does not seem to have a substantial effect on performance. Conversely, using isolated BCAAs orally decreases muscular pain. BCAAs can be found in various supplemental products, such as protein whey, and are frequently mixed with other nutrients, such as carbs. Hence, it is necessary to exercise caution when interpreting the possible advantages of providing athletes with isolated BCAA supplementation in order to reduce muscle soreness and prolong endurance [131,132,133].

### 7.2. Endurance Training Recovery

Optimal recovery after endurance training is contingent upon maintaining proper nutrition. The consumption of carbohydrates and proteins in one’s diet provides the necessary components to improve the replenishment of glycogen and the restructuring of skeletal muscle proteins. Both of these processes are crucial for quickly restoring muscular function and performance. The consensus statement from the Academy of Nutrition and Dietetics (AND), Dietitians of Canada (DC), and the American College of Sports Medicine (ACSM) suggests that for moderate exercise (1 h per day), individuals should consume 5–7 g of carbohydrates per kilogram of bodyweight per day (g/kg/day). For moderate to high intensity exercise (1–3 h per day), the recommended intake increases to 6–10 g/kg/day. Ultra-endurance athletes that have a very high degree of dedication to daily physical activity (4–5 h of exercise at a moderate to high intensity per day) may require a daily intake of 8–12 g per kilogram of body weight [29]. The International Society of Sports Nutrition (ISSN) suggests that athletes should follow a high carbohydrate (CHO) diet, consuming 8–12 g of carbohydrates per kilogram of body weight per day, in order to optimize their glycogen level [134].

When the time available for recovery is limited (less than 8 h), consuming both carbohydrates and proteins soon after exercise can work together to increase the replenishment of glycogen and quickly accelerate the synthesis of muscle protein. However, it should be noted that the ingestion of protein alone can also enhance muscle protein synthesis [135]. In fact, restoring depleted muscle glycogen is a crucial dietary objective, and consuming carbohydrates after exercise remains a widely used and effective food timing strategy to optimize glycogen restoration. In a groundbreaking study by Ivy and colleagues, it was discovered that replenishing muscle glycogen was significantly faster and more thorough when a carbohydrate bolus (2 g/kg of a 25% carbohydrate solution) was consumed within 30 min after a cycling exercise session (lasting 70 min at 68% VO2max, followed by six sets of 2 min intervals at 88% VO2max). This was compared to waiting for two hours after completing the exercise. The restoration of muscle glycogen was found to be 50% quicker and more complete in the former scenario [136]. A recent study carried out by Viribay and colleagues showed that consuming a high amount of carbohydrates (120 g/h) during a mountain marathon may reduce the extent of exercise-induced muscle damage (EIMD) as measured by creatine kinase (CK), lactate dehydrogenase (LDH), and glutamic oxaloacetic transaminase (GOT), as well as the internal exercise load, compared to carbohydrate intake of 60 and 90 g/h [137]. The potential consequences of consuming a larger amount of carbohydrates (120 g/h) compared to the recommended consumption (90 g/h) may result in the development of a more effective approach to reduce exercise-induced muscle damage (EIMD) in physically demanding endurance activities like mountain marathons and ultra-endurance events [137]. In this regard, Loureiro et al. reported that consuming coffee with sweetened milk enhanced the replenishment of muscle glycogen during the 4 h recovery period following intense cycling exercise in comparison to consuming sweetened milk alone. Incorporating coffee into a post-exercise drink that contains sufficient carbs is a successful tactic for enhancing the replenishment of muscle glycogen in cycling competitors who have a limited recovery time (<4 h) or participate in contests involving consecutive and repeated periods of activity [138]. The main type of carbohydrates that are commonly consumed during and after exercise are glucose (polymers). Nevertheless, the ability of the intestinal glucose transport system (SGLT1) may restrict the absorption of glucose in the intestines [139]. Intestinal fructose uptake is primarily dependent on GLUT5 transporters rather than SGLT1 transporters, indicating that it is not regulated by the same transport system. The simultaneous consumption of glucose and fructose can enhance the overall availability of external carbohydrates, resulting in increased rates of external carbohydrate oxidation. Consuming a combination of glucose and fructose can enhance endurance exercise performance when compared to consuming an equivalent amount of glucose alone [140,141]. The use of fructose along with other substances can speed up the process of replenishing glycogen in the liver after exercise. This can be particularly useful when a quick recovery is needed within a time frame of less than 24 h [142].

Historically, endurance athletes have assigned lower importance to protein relative to glucose. Nevertheless, it is crucial for athletes, regardless of whether they are trained in endurance or resistance, to consume enough protein and to do so at the appropriate times. An obsolete model merely adheres to nitrogen balance, which was first devised to avert nutrient insufficiency rather than maximize performance [31]. Athletes necessitate elevated protein consumption above the existing Recommended Daily Allowance (RDA) of 0.8 g/kg/day to attain training adaptations and enhance performance [31]. The American Dietetic Association (AND), Dietitians of Canada (DC), and American College of Sports Medicine (ACSM) all advocate for athletes to consume protein within the range of 1.2–2.0 g per kilogram of body weight per day. Additionally, the International Society of Sports Nutrition (ISSN) suggests a protein intake of 1.4–2.0 g per kilogram of body weight per day [134,143]. However, Churchward-Venne et al. reported that protein consumed following endurance exercise is effectively broken down and assimilated into the bloodstream. These researchers pointed out that the overall balance of protein in the entire body and the integration of amino acids from dietary protein into mitochondrial protein respond to higher protein consumption in a manner that depends on the dosage. Concretely, consuming 30 g of protein is enough to optimize myofibrillar protein synthesis rates when recovering from a single session of endurance exercise [144]. Also, Holwerda et al. added that consuming at least 30 g of protein enhances the pace at which myofibrillar protein synthesis occurs after exercise in older men [145]. However, as will be mentioned later, enhancing daily protein consumption to 1.5 g per kilogram by consuming either whey or soy protein supplements alleviates the decline in field performance that occurs after consecutive speed-endurance training sessions without impacting exercise-induced muscle damage and indications of redox status [146]. Regarding the timing of intake, Trommelen et al. recommended ingesting protein before sleep enhances the rates of protein synthesis in both mitochondria and myofibrils during the overnight recovery period following exercise. Their study specified that there is no difference in the muscle protein synthesis response to whey and casein protein during the night [147]. However, in males who engage in resistance training, the reduction in muscle performance decline and the drop in plasma CK levels after consuming BCAAs supplements are probably insignificant when combined with a diet containing around 1.2 g of protein per kilogram of body weight each day [148].

Regarding antioxidants, the post-exercise repair phase emphasizes the use of antioxidants and anti-inflammatory compounds. An elevated level of antioxidants results in the mitigation of oxidative stress induced by the generation of reactive oxygen species during the inflammatory process [149]. Consequently, the utilization of antioxidants can diminish muscle soreness and aid in recovery in the immediate term. However, excessive doses have also been associated with diminished training advantages in the long run. Levers et al. (2016) discovered that a brief supplementation of 480 mg·day^−1^ of Montmorency powdered tart cherries, combined with a single session of resistance training, resulted in notable improvements in serum markers related to muscle breakdown, physiological stress, and inflammation [150]. Recent research has confirmed that consuming more than 1000 mg of polyphenols derived from fruits on a daily basis can improve muscle regeneration after damage. This is achieved through the antioxidant and anti-inflammatory effects of these compounds [151]. Additional herbal and mushroom supplements with potential benefits for enhancing post-exercise recovery are *Zingiber officinale* [48], *Zingiber officinale + Bixa orellana* L. [152], *Rhodiola rosea* [153]. Furthermore, the adaptogenic, anti-inflammatory, and antioxidant characteristics of ashwagandha make it a promising approach for enhancing recovery and facilitating exercise-induced adaptations [154]. However, further investigation is necessary, although the recommended dosages consist of 300 to 500 mg of aqueous root extract taken twice daily.

### 7.3. Metabolic Conditioning and Strength Training Recovery

Optimal nutrition is essential for the post-exercise recuperation of well-trained athletes, especially following metabolic conditioning or strength sessions. In this regard, prioritizing the consumption of high-quality proteins is crucial for efficient muscle recovery [2,45,155]. Also, carbohydrates are crucial for refilling glycogen stores, and complex alternatives are preferred [156]. Incorporating nutritious fats from sources such as avocados, almonds, and olive oil enhances overall well-being and facilitates the process of recuperation [157]. Additionally, proper hydration is essential, necessitating the replenishment of lost fluids through water or sports drinks in order to maximize performance and facilitate recovery. Recognizing the importance of micronutrients, such as vitamins and minerals, in physiological processes emphasizes the necessity of maintaining a well-balanced diet [158]. It is necessary to mention that personalizing nutrition according to individual requirements is crucial, emphasizing the significance of consulting a nutrition expert or sports dietitian for customized advice in this vital component of athlete health [159]. For instance, the Mediterranean diet (MD) is characterized by a substantial consumption of cereals, vegetables, fruits, nuts, and olive oil. It involves a moderate intake of dairy products, primarily cheese, fish, and poultry, while limiting the consumption of red and processed meats. Studies reported that MD provides athletes with a sufficient supply of carbs, which are suitable for enhancing recovery and optimizing the performance of CrossFit athletes, in addition to bioactive ingredients [160].

Moreover, in a 12-week observational trial, Drobnic et al. [161] examined the impact of krill oil on HS-Omega-3 index levels and plasma choline in relation to performance and recovery following strenuous exercise. The study was centered on the restoration of HS-Omega-3 index levels, the recuperation of plasma choline levels after exercise, and the removal of free radicals following intense physical training. The researchers noted that the simultaneous intake of n-3 PUFAs, choline, and astaxanthin in the form of krill oil did not show a distinct enhancement in athletic performance. Nevertheless, the authors suggest that optimizing the HS-Omega-3 index prior to training, as well as choline and oxidative stress post-training, could be crucial for enhancing athlete performance and facilitating recovery [161].

Additionally, beetroot juice has been studied for its potential in treating hypertension, enhancing physical performance, and aiding in post-exercise recovery [162,163,164]. Research has shown that consuming 250 mL of beetroot juice, three times, with two servings 24 h and 48 h after completing 100 drop jumps, reduced muscle soreness and decreased performance decline in countermovement jumps caused by eccentric exercise. However, it did not seem to have any impact on maximal isometric voluntary contractions, creatine kinase levels, and certain inflammatory markers such as Interleukin 6 (IL-6), tumor necrosis factor alpha (TNF-alpha), and Interleukin 8 (IL-8). Additionally, it seems that taking BCAA as a supplement after intense exercise can create a hormonal milieu that helps decrease strength loss, minimize muscle injury, and promote muscle growth [165]. Also, Correia and colleagues showed that a single-dose of β-hydroxy-β-methylbutyrate free acid (HMB-Fa), generated from leucine, has been found to enhance the recovery of work capacity following high-intensity exercise. Additionally, it may reduce indicators of muscle injury, enhance immediate immunological and hormonal responses, and prevent the loss of lean body mass in catabolic conditions [164,165]. However, according to the results of Tan et al.’s investigation, it is not recommended to simultaneously consume beetroot and pomegranate powder (POM) for the purpose of improving resistance performance. Additionally, the ingestion of BR did not significantly boost performance in the resistance testing battery used in their study [166,167].

Milioni et al. reported that supplementing with β-Alanine during a high-intensity interval training program resulted in an improvement in repeated sprint performance. The augmentation of muscle carnosine levels resulting from β-alanine supplementation may have played a role in reducing central fatigue during repeated sprinting. In summary, incorporating β-alanine supplementation into one’s diet may serve as an effective measure to mitigate fatigue [168]. Regarding sodium bicarbonate, Gough et al. showed that incorporating NaHCO_3_ into the training regimen of high-intensity boxing athletes can effectively enhance acid-base balance recovery and thus increase their performance in boxing [67]. Nevertheless, Malta et al. explicitly stated in their study on cycling that while the administration of NaHCO_3_ frequently caused alkalosis, which suggests the presence of physiological conditions conducive to enhancing performance, it did not have any effect on performance or markers of neuromuscular exhaustion [169]. On the other hand, effect size estimations demonstrate that individuals showed improved performance when they were advised about consuming an ergogenic supplement. These data indicate that the observed performance-enhancing effect of NaHCO3 may be attributed, at least partially, to a placebo effect [169].

Analogous to Bonilla’s four Rs at the beginning of this part, it has been stated that having a balanced nutrition plays a significant role in athlete metabolic or strength training recovery, focusing on quality proteins and complex carbohydrates [108]. Ensuring sufficient hydration and intake of micronutrients is crucial, promoting the need for individualized nutrition guidance. Athletes from numerous disciplines can reap the advantages of adopting the MD. Also, krill oil demonstrated potential for optimizing Omega-3 levels and improving overall performance. Furthermore, beetroot juice, BCAA supplements, and HMB-Fa exhibit promise in decreasing muscle pain and promoting muscular development in various workout scenarios.

## 8. Impact of Dietary Practices on Health

Poor health is significantly influenced by an unhealthy food pattern. The global production, distribution, and consumption of food account for around 25–30% of total greenhouse gas emissions (GHGE), while also affecting other areas of environmental sustainability [69]. Modifying dietary patterns possesses the capacity to enhance public health and contribute to mitigating greenhouse gas emissions (Figure 2) [170]. In the same context, an inadequate diet is the primary contributing factor to mortality and impairment on a global scale. Hunger and malnutrition inflict significant hardship on underprivileged groups worldwide. Concurrently, diet-related cardiometabolic disorders such as coronary heart disease (CHD), stroke, type 2 diabetes, and obesity result in even greater global health costs [171]. Diet-related risk factors also have an impact on other vascular disorders, including peripheral artery disease, chronic renal disease, cognitive decline, heart failure, and atrial fibrillation [172]. Between 2011 and 2030, chronic diseases will result in a total economic loss of USD 17.3 trillion globally. This loss will be due to expenses related to healthcare, decreased productivity, and capital that is lost. Given the significant health and economic challenges they provide, diet-related disorders are currently one of the most pressing concerns [173]. In this regard, over the past few years, there has been a significant change in the eating habits of people worldwide. Simultaneously, the field of nutrition research has made significant progress. In the past two decades, nutrition science has undergone a significant transformation. It has moved away from historical dietary recommendations that were primarily based on cross-national studies, short-term experiments, and animal models. Instead, it now relies on more rigorous evidence from well-designed metabolic studies, prospective cohorts, and randomized clinical trials [171].

**Figure 3:**
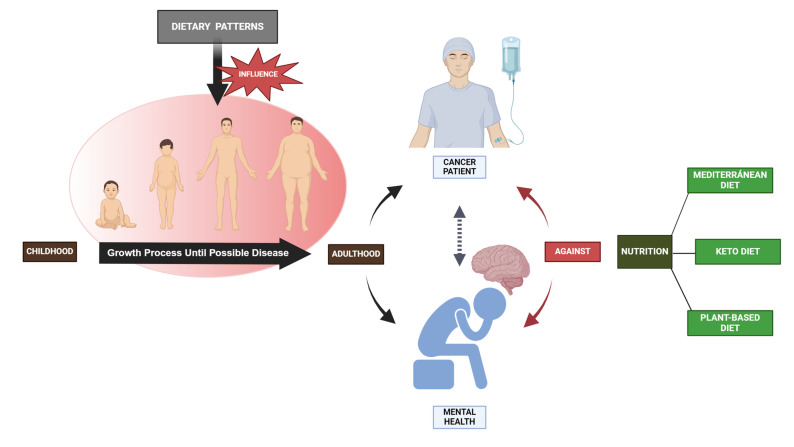
Over the course of a lifetime, poor nutrition can have negative effects on human development, including declining mental health and metabolic issues that can lead to cancer. On the other hand, good nutrition can also help prevent these negative outcomes.

Human nutrition relies on dietary proteins, which are essential. Aside from their role in tissue development, they also impact body composition and control several metabolic processes, as well as feelings of fullness and immune system function [174]. Consuming vegetable protein sources is linked to superior health results, particularly in relation to the cardiovascular system, compared to the use of animal-based products [175]. Also, carbohydrate-rich foods are crucial for maintaining a healthy diet as they supply the body with glucose, which is essential for supporting bodily functions and physical activity [134,135,136,176]. The positive health effects of vegetable protein sources align with their reduced environmental footprint, a crucial factor to be taken into account when formulating an ideal diet. Undoubtedly, the well-being of the Earth is intricately connected to the well-being of human beings [177]. In relation to this, prevention guidelines advocate consuming diets that are abundant in fruits, vegetables, legumes, whole grains, and lean sources of protein, while minimizing or avoiding processed foods, trans-fats, and sugar sweetened beverages. MD, DASH, and plant-based diets have all demonstrated varied degrees of cardioprotective effects and are supported by professional healthcare associations [178]. In the same line, knowing that cancer is a prominent cause of death globally, diet and nutrition have a substantial influence on the initiation and advancement of cancer. In this regard, significant enhancements in reducing cancer development, maintaining a state free of recurrence, and restraining tumor growth were observed in several forms of cancer when following MD, Keto Diet, and plant-based diets (Figure 3) [179]. For instance, greater adherence to the Western-based diet was found to be strongly correlated with an elevated risk of breast cancer (*p* < 0.045). Conversely, the Mediterranean diet was found to have a negative association with the occurrence of breast cancer in pre-menopausal women [180].

Regarding mental health, observational and intervention studies in nutritional psychiatry have provided increasing data on the significance of one’s diet for mental health outcomes throughout one’s life [181,182]. The correlation between nutrition and mental health is reciprocal: the food we consume has an impact on our mental well-being, and our mental well-being influences our dietary choices and consumption quality. In recent decades, there has been a significant rise in the occurrence of mental health issues [82], such as an increasing prevalence of depression, anxiety, cognitive impairments, and sleep difficulties [183]. The role of the diet and its bioactive components in affecting the development of certain diseases has been acknowledged as a modifiable risk factor [184,185]. According to Hossain et al. (2020), there is mounting evidence that suggests specific eating habits can improve brain health [186]. One such pattern is increasing the consumption of fruits, vegetables, and seafood. According to Román et al. (2019), the MD diet has the potential to prevent depression and maintain cognitive function [187]. Fruits, vegetables, whole grains, seafood, and unsaturated fatty acids are usually more prominent on this diet, and moderate alcohol consumption is encouraged on a regular basis. Breast cancer survivors report reduced cancer-related fatigue if their food is of higher quality after diagnosis [188]. In this regard, a new research study has shown links between nutrition, mental health, and cancer, and it found that cancer patients with Mediterranean diet patterns had a lower incidence of depression (Figure 3) [189]. Nevertheless, in order to provide treatment recommendations based on these findings, more research is needed.

When considering the effects of eating habits on health, it is important to acknowledge the environmental factors that arise throughout childhood and the behavioral patterns instilled by our parents [190,191]. Dietary and exercise patterns are established in childhood and tend to continue into adulthood. Consequently, it is crucial to develop healthy lifestyle habits early on in order to promote long-term health and reduce the likelihood of chronic diseases associated with lifestyle choices. The concept of positive parental role modeling suggests that parents have an influence on their child’s eating behavior [192]. This is because children tend to adopt their parents’ food preferences and eating behavior, which, in turn, affects their own dietary behavior and attitudes towards food. For instance, the parents’ attitudes towards healthy eating have been found to positively influence the children’s micronutrient consumption, potentially even more than the parents’ knowledge of what constitutes a healthy childhood [192]. In this regard, Mäkelä et al.’s study contributes to existing knowledge by demonstrating that parents’ perspectives and behaviors about their child’s nutrition and well-being are influenced by their level of health consciousness. The findings also indicate that these factors may additionally impact the nutritional value of the meals that parents offer to their children and the child’s body weight, which can be linked to many long-term diseases [193].

## 9. Enhancing Dietary Habits: Effective Intervention Strategies

The profound significance of nourishment in an athlete’s life cannot be overstated, acting as the foundation for performance, recovery, and overall health [2,14,84,108]. As athletes continually challenge their physical limits, the intricate link between dietary practices and athletic outcomes has taken center stage in sports science [194]. From the meticulous crafting of personalized nutrition plans to educational initiatives, the integration of cutting-edge technology, and psychological tactics, each intervention plays a pivotal role in the comprehensive approach to optimizing athletes’ nutrition [195].

The evolution of athletes’ dietary approaches has witnessed a transformative shift with the rise in personalized nutrition plans [196]. Acknowledging athletes as distinctive individuals with unique body compositions, metabolic rates, and energy needs, recent advances in nutrigenomics unveil the complex relationship between genetic makeup and nutrient response [5,6,13]. Developed collaboratively by sports nutritionists and dietitians, these plans extend beyond macronutrient considerations to cover micronutrient requirements, hydration strategies, and nutrient timing [108,197]. Aligned with factors such as age, gender, training intensity, and individual performance objectives, personalized nutrition plans not only address immediate performance goals but also advocate for long-term health and well-being [198]. By combining scientific precision with personalized care, these plans guide athletes toward a future where optimal performance and individual well-being converge seamlessly [199]. Further, educational programs emerge as transformative measures in optimizing athletes’ dietary practices, seamlessly integrated into training regimens [200]. These comprehensive programs empower athletes with knowledge on essential topics such as macronutrient and micronutrient requirements, hydration strategies, and precise nutrient timing in relation to training and competition schedules [201]. Going beyond information dissemination, these initiatives promote nutritional literacy, transforming athletes from passive recipients of dietary advice into active participants shaping their nutrition [202]. Bridging the gap between theory and practice, athletes acquire practical skills, from deciphering food labels to planning nutritionally balanced meals [203]. In essence, educational programs represent a holistic approach that transcends the conventional athlete–coach dynamic, creating a partnership where athletes become co-architects of their nutritional strategies, catalyzing performance enhancement and fostering a broader narrative of athletes taking charge of their health and performance journey [204].

Moreover, in the age of technological progress, athletes benefit from state-of-the-art tools facilitating precise monitoring and optimization of dietary practices [205]. Advanced body composition analysis technologies, including Dual-Energy X-ray Absorptiometry (DEXA) scans and bioelectrical impedance analysis, deliver accurate insights into an athlete’s muscle mass, fat percentage, and overall body composition [206]. Empowering athletes and their support teams to make real-time adjustments to dietary practices ensures dynamic alignment with specific performance and body composition goals [207]. Beyond immediate impacts on performance, these technological advancements contribute to a proactive approach to well-being, enabling early identification of imbalances and facilitating pre-emptive adjustments to training regimens and nutritional strategies [208]. As these tools evolve, athletes navigate the intricate landscape of sports nutrition with unprecedented precision, ushering in an era where data-driven dietary practices are integral to the holistic enhancement of athletic performance and overall well-being [33,45,87]. Furthermore, interventions focused on nutrient timing have gained prominence, recognizing the critical importance of when nutrients are consumed [208]. Nutrient periodization, aligned with the diverse demands of different training phases and competition schedules, empowers athletes to strategically adjust their nutrient intake for optimized performance outcomes [95]. The triad of pre-, intra-, and post-exercise nutrition assumes pivotal significance in nutrient timing strategies, tailoring nutrition to fuel physical exertion, maintain energy levels, and initiate the recovery process [209]. Synchronizing dietary practices with the ever-changing requirements of an athlete’s training and competition cycles ensures the right nutrients are delivered at the right times, maximizing efficacy for both immediate and long-term performance benefits [210]. Nutrient timing strategies contribute to fatigue prevention, reduction in muscle damage, and enhancements in overall recovery, playing a pivotal role in an athlete’s holistic approach to physical well-being [211]. In addition, athletes, grappling with stress, performance anxiety, and emotional factors shaping their eating habits, benefit from psychological strategies as effective interventions [195,212]. Mindfulness training, cognitive–behavioral interventions, and counselling emerge as indispensable tools, fostering awareness of thoughts and emotions surrounding food, promoting intentional and balanced eating habits, and targeting negative thought patterns for healthier food choices [213]. Beyond mitigating detrimental eating patterns, these psychological strategies empower athletes to navigate the complex relationship between mental states and dietary choices, fostering a sustainable and health-conscious approach to nutrition [214].

Therefore, in the pursuit of athletic excellence, interventions to improve dietary practices stand as indispensable components of a holistic approach to sports performance. Personalized nutrition plans, educational programs, technological advancements, nutrient timing strategies, and psychological interventions collectively contribute to fostering a culture of informed and health-conscious dietary choices among athletes. As the field of sports science continues to evolve, these interventions serve as valuable tools in optimizing nutrition, ensuring athletes are not only physically prepared but also nutritionally equipped to excel in their chosen sports. Through a combination of science, education, and individualized strategies, athletes can harness the power of nutrition to enhance their performance, recovery, and overall well-being.

## 10. Case Studies of Successful Dietary Strategies of Elite Athletes

The adaptability to training is intricately linked with the interplay of training intensity, volume, and frequency, as underscored by Mujika et al. (2014) [92]. This dynamic interconnection between nutrition and exercise is critical in shaping training adaptations. Notably, dietary variations exert a significant influence on training adaptation, highlighting the diverse possibilities within periodized nutrition or nutritional training. However, it is important to recognize that not all practices within this domain are equally substantiated by scientific evidence. Acknowledged practices include training with varying carbohydrate availability, supplement use, and enhancement of intestinal function.

Athletes and trainers should be mindful that there are no miraculous shortcuts in nutritional periodization, which should be strategically targeted towards specific objectives. Combining different methods can effectively optimize nutritional training, as suggested by Jeukendrup (2017) [93]. The ultimate aim of nutritional periodization is to facilitate the body’s adaptation to training, taking into consideration factors such as age, sex, physical condition, prior nutritional state, diet, and specific training factors like intensity, volume, timing, and rest periods. These elements are crucial in determining the effectiveness of nutritional training in fostering training adaptations, according to Kerksick et al. (2017) [94]. Given the vast diversity of athletic events, each with unique bioenergetic, biomechanical, and structural demands, a broad spectrum of periodized nutrition combinations is required. Therefore, implementing nutritional training strategies for proteins, supplements, or carbohydrates, and closely monitoring their outcomes is essential.

The plasticity of skeletal muscle is a key factor in how it adapts its metabolism to different training loads and nutrient availabilities. The variability in macronutrient intake leads to changes in the availability of substrates and hormones in the blood, which, in turn, affects muscle and insulin-sensitive tissues. This interaction between the available nutrients and physical exercise plays a critical role in determining the body’s ability to activate or inhibit various biochemical pathways, thereby influencing the effectiveness of training adaptations. This relationship is crucial in understanding how dietary choices impact the physiological responses to training, as highlighted by Hawley et al. (2011) [96].

In the context of developing endurance, skeletal muscle demonstrates notable training adaptations, which can be significantly enhanced through the application of nutritional periodization. Specifically, adopting a strategy that involves consuming fewer carbohydrates during strategically planned periods, while increasing protein intake, is believed to foster greater training adaptations, as illustrated by Close et al. (2016) [97]. Furthermore, the response to endurance training can be amplified through various factors like glycogen depletion, caloric restriction, and oxidative stress. These factors elevate the molecular level stress, consequently increasing the quantity and activity of peroxisome proliferator-activated receptor gamma coactivator 1-alpha (PGC-1α). This activation plays a pivotal role in inducing primary adaptive responses to resistance exercise, such as mitochondrial biogenesis, angiogenesis, and enhanced fat oxidation, highlighting the importance of PGC-1α in the overall training adaptation process. This suggests that the activation of PGC-1α can be effectively modulated through targeted nutritional training strategies, as per Baar [98].

Ultra-endurance athletes, while sharing many training habits with traditional endurance athletes, tend to have a higher prevalence of vegan or vegetarian diets, as noted by Nikolaidis et al. [99]. These athletes also have a higher carbohydrate intake, which is crucial for exogenous carbohydrate oxidation and mitigating gastrointestinal discomfort during ultra-endurance events. In terms of pre-competition nutrition, there is an emphasis on fat utilization as fuel during exercise. The nutritional adaptations required for endurance training are diverse and specific to the type of training undertaken. For instance, training at moderate altitudes (approximately 1600–2400 m) introduces unique challenges, including the risks associated with insufficient energy availability and the need for increased iron intake to avoid compromising training adaptations. Additionally, oxidative stress from altitude training should be addressed through dietary antioxidants, rather than supplementation, as suggested by Stellingwerff et al. [100]. Combining these metabolic, cardiovascular, and muscular adaptations with appropriate nutrition and supplementation is key to enhancing performance for endurance athletes, as highlighted by Earnest et al. [101].

In the realm of high-intensity sports, the use of dietary supplements for enhancing anaerobic performance garners considerable interest. The energy required for anaerobic processes is fulfilled through adenosine triphosphate (ATP), with its production relying on muscular phosphocreatine (PCr) content, buffering capacity, and the volume of contracting muscle mass. While the latter two aspects can be augmented through anaerobic training, PCr remains unaffected by such training. Significant scientific evidence supports the benefits of dietary ergogenic aids like creatine, sodium bicarbonate, and beta-alanine in augmenting anaerobic capacity, as noted by Sahlin [102]. However, the impact of these supplements on athletic performance can be inconsistent. For instance, a study revealed that five-day supplementation with creatine monohydrate, a commonly used aid, did not enhance the anaerobic power in trained wrestlers [103]. Sodium bicarbonate, administered at 0.3 g/kg of body mass, appears to be an effective ergogenic aid, but it is often associated with side effects like intestinal discomfort. Additionally, beta-alanine supplementation has been reported to cause paresthesia after a single intake. As a result, combining sodium bicarbonate and beta-alanine may be a more effective strategy, as suggested by Lancha Junior et al. [104].

Despite the strong scientific support for the combined use of bicarbonate and beta-alanine, as well as creatine with beta-alanine, in enhancing performance in high-intensity exercises, the benefits might vary depending on the specific type of exercise and the athlete’s training state. This indicates that the effectiveness of these supplements can be contingent upon individual athletic profiles and the nature of the exercise being undertaken. Consequently, there is a need for further research to unravel the intricacies of these synergistic effects and to tailor supplement strategies more effectively for individual athletes. This approach will aid in optimizing performance while also considering the unique physiological responses of each athlete [105].

## 11. Future Directions in Sports Nutrition Research

The trajectory of sports nutrition research is increasingly aligning with the paradigm of personalized nutrition, recognizing the unique interplay between diet and athletic performance. This shift acknowledges the diversity in individual responses to dietary interventions, influenced by a variety of factors including genetics, microbiome composition, and metabolic rate. The burgeoning field of nutrigenomics, which explores the interaction between nutrition and genes, is set to play a pivotal role. By understanding genetic variations that affect nutrient metabolism and dietary responses, researchers can develop more nuanced dietary strategies to optimize body composition, enhance recovery, and elevate overall athletic performance.

In parallel, the role of the gut microbiome in sports performance is gaining unprecedented attention. The gut microbiota, with its profound influence on nutrient metabolism, immune function, and mental well-being, is a critical determinant of athletic performance [215]. Future research is expected to dissect how dietary interventions can modulate the gut microbiome, thereby influencing performance outcomes. This exploration is particularly relevant in the context of personalized nutrition, as the gut microbiome varies significantly among individuals. Furthermore, the integration of advanced technology and data analytics is another frontier. Wearable technology and mobile applications are revolutionizing the way dietary intake, physiological responses, and performance metrics are monitored and analyzed in real time [216]. Coupled with the power of machine learning and artificial intelligence, these tools can synthesize vast amounts of data to provide tailored nutritional recommendations, aligning with the unique physiological and performance needs of each athlete.

This evolution in sports nutrition research is not isolated but is deeply interconnected with various aspects of athletic performance and health. For instance, understanding the relationship between body composition and performance is crucial in personalizing dietary strategies. Different sports have distinct nutritional requirements, and evaluating current dietary practices of athletes provides a baseline for personalized interventions. Factors affecting nutritional choices, such as cultural influences and accessibility, must be considered in developing tailored dietary plans. Moreover, the interplay between nutrition and training adaptations is a critical area of focus. Personalized nutrition strategies can significantly influence how athletes adapt to training stimuli, impacting muscle recovery, energy utilization, and overall performance efficiency. Optimal nutrition for recovery is another critical aspect, where personalized dietary interventions can expedite recovery processes, reduce injury risk, and enhance training outcomes.

The impact of dietary practices on health extends beyond performance enhancement to encompass long-term health and well-being. Personalized nutrition strategies must balance performance goals with the prevention of nutrition-related chronic diseases. In this context, interventions to improve dietary practices are essential. These interventions, grounded in personalized nutrition, can range from educational programs to the use of advanced technologies for dietary monitoring and feedback. Then, the future of sports nutrition research is a tapestry woven with the threads of personalized nutrition, advanced technology, and a deep understanding of the multifaceted nature of diet, health, and athletic performance. This holistic approach promises not only to enhance the performance of athletes but also to contribute to their overall well-being and long-term health. As this field evolves, it will undoubtedly offer groundbreaking insights and innovative strategies that will redefine the landscape of sports nutrition.

## 12. Practical Applications

Personalized nutrition plans for athletes: Sports dietitians and nutritionists can leverage the insights from this review to develop individualized nutrition plans. These plans should consider the athlete’s specific sport, body composition goals, training schedule, and any unique physiological or metabolic needs. For instance, endurance athletes may require diets higher in carbohydrates for energy, while strength athletes might need increased protein for muscle repair and growth.

- Enhanced training programs: Coaches and trainers can integrate nutritional strategies into training regimens. Understanding the role of nutrition in muscle recovery and energy metabolism can help in designing training programs that align with the athlete’s dietary intake. This approach ensures that athletes are adequately fueled for their workouts and recover more effectively, reducing the risk of overtraining and injury.
- Use of wearable technology for monitoring: Athletes and their support teams can employ wearable technology to monitor dietary intake, physiological responses, and performance metrics. These real-time data can be used to make immediate adjustments to dietary plans, ensuring optimal performance and recovery.
- Nutrigenomics for personalized dietary strategies: The burgeoning field of nutrigenomics explores the interaction between nutrition and genes. Developing personalized dietary strategies based on understanding genetic variations that affect nutrient metabolism and dietary responses.
- Educational workshops for athletes and coaches: Organizing workshops and seminars on sports nutrition can be invaluable. These sessions can cover topics like the importance of macronutrients and micronutrients, hydration strategies, and the timing of nutrient intake. Educating athletes and coaches about the impact of nutrition on performance and health can lead to better dietary choices.
- Gut microbiome analysis for dietary optimization: Given the emerging role of the gut microbiome in athletic performance, offering gut microbiome analysis can be a novel service for athletes. This analysis can provide insights into how an athlete’s diet might be optimized to improve nutrient absorption, immune function, and even mental well-being.
- Recovery nutrition strategies: Developing specific nutritional strategies for recovery post-training and competition can significantly enhance an athlete’s ability to return to peak performance quickly. This includes not only the right balance of proteins and carbohydrates but also incorporating anti-inflammatory foods and adequate hydration.
- Nutrition for injury prevention and management: Utilizing nutrition as a tool for injury prevention and management can be a key application. Adequate intake of nutrients like calcium and vitamin D for bone health, omega-3 fatty acids for inflammation, and antioxidants for tissue repair can be integral parts of an athlete’s diet.
- Customized supplement plans: Based on individual needs and deficiencies identified through regular health and nutritional assessments, customized supplement plans can be developed. These should be used judiciously and in compliance with anti-doping regulations.
- Mental health and nutrition: The role of diet in mental health can be addressed, particularly for athletes under high stress or those recovering from injuries. Incorporating foods that support mental well-being, such as those rich in omega-3 fatty acids and B vitamins, can be part of a holistic approach to athlete health.
- Community and team-based nutrition programs: Implementing team-based nutrition programs, especially in team sports settings, can foster a supportive environment for making healthier food choices. These programs can include group education sessions, cooking workshops, and team meals planned by a sports nutritionist.

## 13. Conclusions

The intricate nexus of dietary practices, body composition, and athletic performance constitutes a complex and evolving domain within sports science. Contemporary research underscores the imperative for personalized nutrition strategies, meticulously tailored to accommodate the distinct physiological requirements and performance objectives of each athlete. This bespoke approach is instrumental in optimizing athletic performance, underscoring the necessity of individualized dietary interventions. Advancements in technology and scientific inquiry are pivotal in refining sports nutrition. The integration of these advancements facilitates the development of nuanced dietary interventions, enhancing their precision and efficacy. This evolution in sports nutrition is not confined to the realm of elite athletics; it extends its benefits to all individuals engaged in physical activities, aiming to augment their health and performance metrics. The potential of this evolving field to revolutionize sports nutrition and elevate athletic prowess is substantial. As our comprehension of the interrelationships between diet, body composition, and performance deepens, we stand on the cusp of transformative changes in sports nutrition practices.
